# Short-term evaluation of motor and sensory nerve conduction parameters in COVID-19-associated peripheral neuropathy patients

**DOI:** 10.1186/s43168-023-00189-3

**Published:** 2023-03-10

**Authors:** Ahmad M. Shaddad, Aliaë A. R. Mohammed Hussein, Amal Mohamed Aly Tohamy, Waleed Gamal Elddine Khalil

**Affiliations:** 1 Chest Department, Faculty of Medicine, Assiut University, Assiut, 71515 Egypt; 2 Neuropsychiatry Department, Faculty of Medicine, Assiut University, Assiut, 71515 Egypt

**Keywords:** COVID-19, Nerve conduction studies, Peripheral neuropathy, Complication, Motor and sensory function, Nerve conduction velocity, Nerve conduction amplitude, Nerve conduction latency, *F* wave latency

## Abstract

**Background:**

Severe acute respiratory syndrome coronavirus 2 (SARS‐COV‐2) is mostly associated with upper and lower respiratory tract manifestations. However, coronavirus disease 19 (COVID-19) can result in a wide range of other systemic symptomatology, including neuropsychiatric, psychological, and psychosocial impairments. Literature regarding neurological compromise, including neuropathy and sensory and motor affection associated with COVID-19, is still limited.

This study aims to evaluate the sensory, motor neuropathy, and secondary neurological impairment among patients with mild to moderate coronavirus disease associated with peripheral neuropathy within 1 month.

**Methods:**

Forty participants, including 20 mild to moderate COVID-19 patients with peripheral neuropathy and 20 age and gender-matched healthy volunteers, were recruited in this case/control study. Laboratory evaluation focused on C-reactive protein (CRP) and D-dimer levels. Oxygen saturation for all participants was recorded. The neurophysiological study included motor nerve study, sensory nerve study, and *F* wave study for upper and lower limbs were done.

**Results:**

The two groups were similar regarding baseline data. Neurological symptoms’ onset in the COVID-19 group ranged from 4 to 24 days. Levels of CRP and D-dimer levels were significantly higher in patients versus the control group. Motor nerve conduction (MNC) amplitude and latency for the median nerve were significantly compromised among the COVID-19 group. The MNC latency and *F* wave latency for the posterior tibial nerve were significantly higher in the COVID-19 group. The CRP and D-dimer levels were associated with a significant positive correlation with a latency of median nerve MNC, sensory nerve conduction (SNC), and f-wave; latency of MNC and *F* wave of the posterior tibial nerve; and SNC latency for sural nerve.

**Conclusion:**

neurological involvement can occur in mild to moderate cases of SARS-COV-2 infection and add to the burden of the disease. Neurological symptoms in the course of COVID-19 disease should be interpreted cautiously, and appropriate diagnosis, including nerve conduction studies and management, should be considered.

**Trial registration:**

ClinicalTrials.gov. NCT05721040.

## Introduction

Severe acute respiratory syndrome coronavirus 2 (SARS-CoV-2), which first emerged in Wuhan, China, can result in a wide variety of symptomatology ranging from asymptomatic infection up to pneumonia, respiratory failure, or even death [1].

Respiratory involvement is the prevalent form of COVID-19 disease; however, growing evidence suggests neurological manifestations to be a direct consequence and even the presenting manifestation in a large proportion of patients [2]. Cerebrovascular diseases, including stroke, encephalitis, neuropathy, visual pathway abnormality, and Guillain-Barré syndrome, were recorded. Other common features encountered early in the course of infection were loss of smell (anosmia) and taste (ageusia) [3–6].

Several theories emerged to explain neurological manifestations, including direct neuroinvasion by the coronavirus, [7, 8] explained through the activation of the angiotensin-converting enzyme 2 (ACE-2) receptor expressed in both capillary and neuronal endothelium [9]. Dysregulated immune response, [10, 11] hypoxemia, multiorgan involvement, and increased prothrombin time and coagulopathy are other explanations [12, 13].

This study aims to evaluate the pattern of motor and sensory, and neurological affections in mild to moderate COVID-19 with associated peripheral neuropathy patients and explore the relationship between inflammatory status and neurological deficits.

## Patients and methods

### Study design and participants

This prospective case–control study was conducted in the Chest Department, Faculty of Medicine, Assiut University, and the Neurology Department, Faculty of Medicine, Assiut University, between October 2021 and April 2022. Twenty RT-PCR-confirmed mild to moderate COVID-19 patients with peripheral neuropathy presented within 1 month of acute disease at outpatient clinics of the Chest and Neurology Departments were recruited and twenty healthy age and gender-matched volunteers.

### Inclusion and exclusion criteria

All patients with confirmed RT-PCR COVID-19 infection with mild to moderate severity based on the Egyptian Ministry of Health (MOH) protocol (version 1.4, November 2020) with neurological symptoms suggestive of peripheral neuropathy were eligible for participation in the current study. Exclusion criteria were patients presenting with neurological manifestations other than peripheral neuropathy, such as stroke, cerebral hemorrhage, encephalitis, or meningitis. Patients with any medical condition or comorbidities that may affect the result of the study as diabetes, arthritis, and carpel tunnel syndrome were excluded. Severe COVID-19 patients with a critical condition that needs hospitalization and ventilatory support were also excluded from the study.

### Clinical and laboratory assessment

All patients were subjected to intense history taking, including the onset of the COVID-19 infection and the start of neurological symptoms, careful clinical examination, and neurological assessment. All patients and control groups had oxygen saturation, CPR, and D-dimer measurement at the time of the study.

### Neurophysiological study

All studies were conducted using A Nihon Kohden Machine model 9400 (Tokyo, Japan).

*The motor nerve study* was performed in the median and posterior tibial nerve to assess the motor function of the upper and lower limbs. A standard procedure with concentric needle electrodes is used to assess motor nerve conduction velocity. Supramaximal intensity stimulation with a duration of 0.2 ms with a rate of one per second was performed. Compound motor action potential (CMAP), motor nerve conduction velocity (MNCV), and nerve conduction latency were recorded.

*The sensory nerve study* was done for the median and sural nerves to estimate the peripheral neurological function of both upper and lower limbs. The analysis was performed using stimulating ring electrodes placed over the middle and proximal phalanxes of the second and third fingers. In contrast, the recording electrode is placed over the palm in a position 1–2 cm proximal to the proximal crease of the palm. The sensory nerve conduction study of the sural nerve was performed by placing surface recording electrodes over the posterior aspect of the calf at a point between the middle and lower thirds of the leg, just lateral of the midline. Stimulating electrodes are placed behind and below the lateral malleolus over the sural nerve. Sensory motor nerve conduction amplitude, latency, and velocity were recorded.

*F wave study* was performed for the upper limb in the median nerve by placing the recording electrode at the abductor pollices brevis muscle. In contrast, the recording electrodes were placed two cm distal near the tendon insertion. For testing the *F* wave in the posterior tibial nerve for the lower limb, recording electrodes were placed over the abductor hullucis muscle, and the stimulating electrodes were placed at the malleolus. For *F* wave recording, a supramaximal stimulus was performed, ten stimuli were given, and their average was recorded.

For all parameters of neurophysiological studies, the values below the 95th percentile or ± 2SD of control are considered abnormal. Diffuse axonal neuropathy was diagnosed by the reduction of CMAP amplitude with standard shape and duration and with normal or minimal disturbance of nerve conduction velocity. In contrast, diffuse demyelinating neuropathy was diagnosed by increased nerve conduction latency, preserved amplitude, and average nerve conduction velocity.

*Statistical analysis and sample size* were performed using the SPSS program (version 20, IBM and Armonk, New York). The Mann–Whitney test was used for continuous data, while chi^2^ test compared nominal data. Different correlations of continuous variables in the study were assessed with Spearman’s correlation. The sample size was estimated by Open Epi V.3.01 computer program.

### Ethical considerations

All participants subjected to the study were asked to apply informed written consent; the research’s nature, procedures, and possible side effects were clearly explained. The Faculty of Medicine, Assiut University’s ethical committee approved the study protocol under the Declaration of Helsinki.

## Results

In the current study, we recruited twenty COVID-19 patients with confirmed diagnoses by RT-PCR with neurological symptoms and twenty genders and age-matched healthy volunteers.

The demographic characteristics of the patients and the control group are shown in (Table 1). There was no significant difference between the two groups regarding age, gender, education, smoking, and residence. The mean onset of neurological symptoms is 11.95 ± 5.9 days and ranges from 4 to 24 days.

**Table 1:** Demographic data of the COVID-19 patients with peripheral neuropathy and control group (*n* = 40)

	**COVID-19 group** ***N* = 20**	**Control group** ***N* = 20**	***P* value**
**Gender**			
Male	17 (85%)	16 (80%)	0.50
Female	3 (15%)	4 (20%)	
**Age** (years)			
Mean ± SD	45.05 ± 11.05	42.35 ± 11.05	0.745
Range	(21–68)	(20–59)	
**Smoking**			
Smoker	8 (40%)	9 (45%)	0.465
Non-smoker	11 (55%)	8 (40%)	
X smoker	1 (5%)	3 (15%)	
**Education**			
Literate	15 (75%)	17 (85%)	0.347
Illiterate	5 (25%)	3 (15%)	
**Residence**			
Urban	13 (65%)	16 (80%)	0.480
Rural	7 (35%)	4 (20%)	
**The onset of neurological symptoms (Range)**	11.95 ± 5.90 (4–24)	–-	

Data expressed as frequency (percentage) and mean (SD). *P* value was significant if < 0.05

There were significant differences between the patient group and the control group regarding; CRP level, where the mean value in the COVID-19 group was 39.7 ± 7.46 and 6.3 ± 2.15 in the control group with a *p* value of < 0.001, D-dimer with a mean value in the patients’ group of 983.6 ± 324.88 and the control group of 315.3 ± 63.03 with a *p* value of < 0.001 and oxygen saturation in the COVID-19 group with the mean of 96.95 ± 1.35 and the control group of 98.5 ± 0.61 with a *p* value of 0.009 (Table 2).

**Table 2:** Laboratory data and oxygen saturation level of the COVID-19 patients with peripheral neuropathy and control group (*n* = 40)

	**COVID-19 group** ***N* = 20**	**Control group** ***N* = 20**	***P* value**
CRP	39.7 ± 7.46	6.30 ± 2.15	< 0.001*
D-dimer	983.6 ± 324.88	315.30 ± 63.03	< 0.001*
SpO_2_	96.95 ± 1.35	98.5 ± 0.61	0.009*

Data expressed as frequency (percentage) and mean (SD). *P* value was significant if < 0.05

*CRP* C-reactive protein, *CRP* C-reactive protein, *SpO*_*2*_ Saturation of peripheral oxygen

Regarding the nerve conduction study in both groups, there was a significant difference between the two groups in median nerve motor nerve conduction amplitude (MNCA) with a mean of 8.88 ± 2.6 and 9.04 ± 2.12 in the control group with a *P* value of 0.039. There was a significant statistical difference between the two groups in median nerve motor nerve conduction latency (MNCL) with a mean of 5.1 ± 1.34 and 3.29 ± 0.64 in the control group with a *P* value of < 0.001. There was a significant statistical difference between the two groups in posterior tibial nerve motor nerve conduction latency (MNCL) with a mean of 3.99 ± 0.99 and 3.06 ± 0.61 in the control group with a *P* value of 0.007. There was a significant statistical difference between the two groups in Posterior tibial nerve *F* wave latency with a mean of 52.28 ± 4.95 and 47.25 ± 3.17 in the control group with a *P* value of 0.002 (Table 3).

**Table 3:** Electrophysiological measurements of the COVID-19 group with the peripheral neuropathy and control group (*n* = 40)

	**COVID-19 group** ***N* = 20**	**Control group** ***N* = 20**	***P* value**
MNC amplitude (mV)	8.88 ± 2.60	9.04 ± 2.12	
Median nerve	5.12–13.5	6.43–17.10	0.039*
MNC latency (ms)	5.1 ± 1.34	3.29 ± 0.64	
Median nerve	2.59–7.45	2.16–4.78	< 0.001*
MNC velocity (m/s)	58.06 ± 6.4	58.18 ± 7.30	
Median nerve	42.40–69.30	45.92–70.9	0.220
SNC amplitude (mV)	21.18 ± 8.76	21.19 ± 5.94	
Median nerve	11.5–45.4	12.45–38	0.091
SNC latency (ms)	4.33 ± 0.91	3.47 ± 0.72	
Median nerve	3.08–6.20	2.76–5.23	0.305
SNC velocity (m/s)	51.7 ± 5.62	51.99 ± 6.84	
Median nerve	42.9 – 59.24	41.29–69.2	0.753
*F* wave latency (ms)	33.03 ± 4.59	30.14 ± 6.16	
Median nerve	26.6–39.3	24.56–44.56	0.389
MNC amplitude (mV)	12.30 ± 2.15	12.84 ± 2.70	
Post Tibial nerve	7.39–17.53	8.24–19.1	0.366
MNC latency (ms)	3.99 ± 0.99	3.06 ± 0.61	
Post Tibial nerve	2.25–5.6	2.29–4.22	0.007*
MNC velocity (m/s)	56.6 ± 5.44	57.05 ± 5.24	
Post Tibial nerve	49.2–69.1	51.2–68.3	0.703
SNC amplitude (mV)	28.1 ± 4.44	28.25 ± 4.44	
Sural nerve	21.2–35.3	21.3–36.2	0.956
SNC latency (m/s)	3.81 ± 0.6	3.01 ± 0.59	
Sural nerve	2.68–4.70	2.11–3.98	0.760
SNC velocity (m/s)	56.7 ± 8.25	57.15 ± 6.05	
Sural nerve	43.1–76.2	47.1–68.4	0.281
*F* wave latency (ms)	52.28 ± 4.95	47.25 ± 3.17	
Post Tibial nerve	43.2–61.6	42.3–52.1	0.002*

Data expressed as frequency (percentage) and mean (SD). *P* value was significant if < 0.05

*MNC* Motor nerve conduction, *SNC* Sensory nerve conduction

We correlated the laboratory data and age with the neurophysiological parameters. There was a positive correlation between CRP level and median nerve (MNCL) with *r* = 0.787, *p* value < 0.001; median nerve (SNCL) with *r* = 0.668, *p* value < 0.001; median nerve *F* wave latency with *r* = 0.386, *p* value < 0.014; posterior tibial nerve (MNCL) with *r* = 0.611, *p* value < 0.001; sural nerve (SNCL) with *r* = 0.624, *p* value < 0.001; and posterior tibial nerve *F* wave latency with *r* = 0.544, *p* value < 0.001.

There was a positive correlation between D-dimer level and median nerve (MNCL) with *r* = 0.702, *p* value < 0.001; median nerve (SNCL) with *r* = 0.590, *p* value < 0.001; median nerve *F* wave latency with *r* = 0.418, *p* value < 0.007; posterior tibial nerve (MNCL) with *r* = 0.493, *p* value < 0.001; sural nerve (SNCL) with *r* = 0.657, *p* value < 0.001; and posterior tibial nerve *F* wave latency with *r* = 0.557, *p* value < 0.001.

There was a negative correlation between oxygen saturation and median nerve (SNCL) with *r* =  − 0.542, *p* value < 0.001; median nerve *F* wave latency with *r* =  − 0.364, *p* value < 0.021; posterior tibial nerve (MNCL) with *r* =  − 0.565, *p* value < 0.001; sural nerve (SNCL) with *r* =  − 0.640, *p* value < 0.001; posterior tibial nerve *F* wave latency with *r* =  − 0.546, *p* value < 0.004; and positive correlation between oxygen saturation and median nerve (MNCL) with *r* = 0.619, *p* value < 0.001; median nerve (SNCV) with *r* = 0.328, *p* value 0.039; and posterior tibial nerve (CMAP) with *r* = 0.312, *p* value 0.049) (Table 4).

**Table 4:** Correlation of age, CRP value, D-dimer value, and SpO_2_ with nerve conduction study results in COVID-19 cases with peripheral neuropathy (*n* = 20)

	**Age**		**CRP**		**D dimer**		**SpO**_**2**_	
	***r***	***P***	***R***	***P***	***R***	***P***	***r***	***P***
MNC amplitude								
Median nerve	0.055	0.735	− 0.079	0.629	− 0.143	0.379	0.149	0.358
MNC latency								
Median nerve	− 0.089	0.585	**0.787**	< 0.001*	**0.702**	< 0.001*	**0.619**	< 0.001*
MNC velocity								
Median nerve	− 0.128	0.431	− 0.049	0.766	− 0.048	0.769	0.008	0.959
SNC amplitude								
Median nerve	− 0.007	0.966	− 0.212	0.189	0.113	0.489	0.112	0.492
SNC latency								
Median nerve	− 0.003	0.988	**0.668**	< 0.001*	**0.590**	< 0.001*	− **0.542**	< 0.001*
SNC velocity								
Median nerve	0.061	0.710	− 0.082	0.616	− 0.121	0.457	0.328	0.039*
*F* wave latency								
Median nerve	0.137	0.399	**0.386**	0.014*	**0.418**	0.007*	− **0.364**	0.021*
MNC amplitude								
Post-tibial nerve	− 0.300	0.060	− 0.051	0.757	− 0.076	0.642	**0.312**	0.049*
MNC latency								
Post = tibial nerve	− 0.002	0.992	**0.611**	< 0.001*	**0.493**	0.001*	− **0.565**	< 0.001*
MNC velocity								
Post-tibial nerve	− 0.015	0.929	− 0.068	0.676	− 0.212	0.188	0.197	0.223
SNC amplitude								
Sural nerve	− 0.149	0.358	− 0.111	0.494	− 0.065	0.690	0.132	0.417
SNC latency								
Sural nerve	0.018	0.914	**0.624**	< 0.001*	**0.657**	< 0.001*	− **0.640**	< 0.001*
SNC velocity								
Sural nerve	0.202	0.211	0.004	0.980	0.005	0.978	0.002	0.98
*F* wave latency								
Post-tibial nerve	0.046	0.777	**0.544**	< 0.001*	**0.557**	< 0.001*	− **0.446**	0.004*

Correlation for variables in the study was determined with spearman’s correlation. *r* correlation coefficient rho. *P* value: was significant if < 0.05

*CRP* C-reactive protein, *SpO*_*2*_ Saturation of peripheral oxygen, *MNC* Motor nerve conduction, *SNC* Sensory nerve conduction

**Fig. 1:**
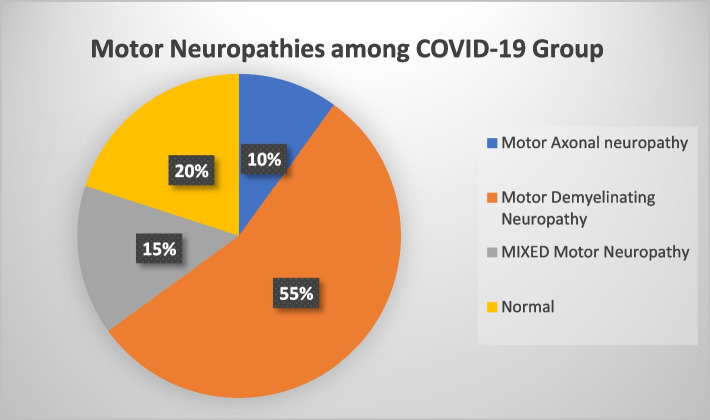
Frequency and types of motor neuropathies among COVID-19 groups (*n* = 20)

**Fig. 2:**
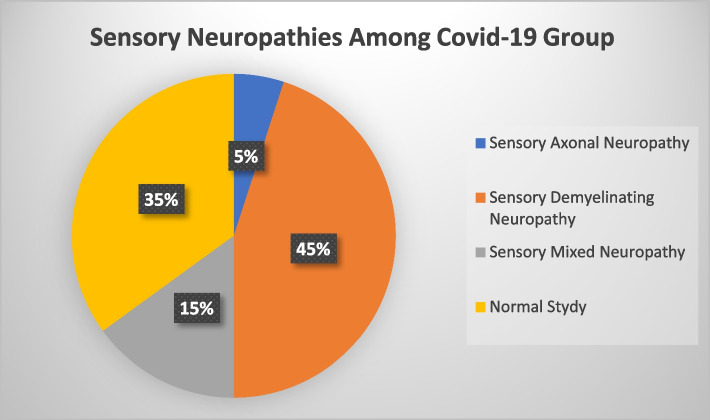
Frequency and types of sensory neuropathies among COVID-19 groups (*n* = 20)

Stratification of the motor nerve conduction study of the COVID-19 group revealed that 10% of patients had motor axonal neuropathy, 15% mixed motor neuropathy, and 55% demyelinating motor neuropathy, while 20% of patients with a normal study (Fig. 1). Assessment of the sensory nerve conduction study showed that 5% of patients had sensory axonal neuropathy, 15% mixed sensory neuropathy, and 45% sensory demyelinating neuropathy, while 35% of patients with a normal study (Fig. 2).

## Discussion

In the current study, we enrolled twenty mild to moderate COVID-19 patients with peripheral neuropathy and compared them with 20 age and sex-matched healthy controls. Neurophysiological studies revealed a significant difference between the two groups in compound motor action potential and motor nerve conduction latency of the median nerve and motor nerve conduction latency of the posterior tibial nerve and *F* wave latency of the posterior tibial nerve. There was a positive correlation between oxygen saturation and SNC velocity of the median nerve, the MNC amplitude of the post-tibial nerve. There was a strong negative correlation between SpO_2_ and MNC latency, SNC latency, and *F* wave latency of the median nerve. Also, strong negative correlation between SpO_2_ and MNC latency of the posterior tibial nerve, SNC latency of the sural nerve, and *F* wave latency of the posterior tibial nerve.

There was a strong positive correlation between D-dimer level and CRP level with MNC, SNC, and *F* wave latencies of the median nerve, MNC and *F* wave latencies of the posterior tibial nerve, and SNC latency of the sural nerve. Motor axonal neuropathy was observed in 10% of patients; motor demyelinating neuropathy was observed in 55%, and mixed motor neuropathy in 15%. Sensory axonal neuropathy was observed in 5% of patients; demyelinating sensory neuropathy was observed in 45%, and mixed sensory neuropathy in 15%.

Neurological affection is observed throughout the course of the COVID-19 infection. A study by Mao and his colleagues on 214 COVID-19 patients observed the presence of neurological manifestations in 36% of the study group [14]. Although SAR-COV-2 is not documented to be present in CSF of affected patients during the course of COVID-19 infection; there is a strong association between the presence of neurological symptoms and SARS-COV-2 infection [15].

In an attempt to explain the pathophysiology of neurological affection in SARS-COV-2 infection; the international human cell atlas community has reported increased expression of two key co-receptors during SARS-COV-2 infection, namely ACE2 and TMPRSS2 co-receptors [16]. Another theory is the para-infectious neurological syndrome due to SARS-COV-2 infection [17].

A possible etiological explanation is the host immune response to SARS-COV-2 and the development of cytokine storms [18]. Peripheral nervous system and muscle affection is common through the course of SARS-COV-2 infection with a median of 7 days (range 7–24) [19].

In agreement with our study, EL-Leithy et al. enrolled 60 COVID-19 patients in 3 groups (20 mild, 20 moderate, and 20 severe cases) with a significant difference in upper and lower nerve conduction study between the patient groups and the control group, which were significantly correlated to the CRP level [20].

Elshebawy et al. enrolled 42 patients; 23 of them were in the first wave of COVID-19, and 19 were in the second wave; the characteristics of the study groups showed the presence of acute inflammatory demyelinating neuropathy in 47.8% of the 1st wave patients, and 73.7 in the 2nd wave patients and the demyelinating with secondary axonal affection in 13% of the 1st wave patients and 10.5% of 2nd wave patients [21].

Ellul and colleagues using data available up to May 19, 2020. COVID-19 cases based on Johns Hopkins COVID-19 Dashboard demonstrated that peripheral neuropathy is present in 0.05% of COVID-19 patients [16].

In a Cohort of Egyptian Patients with COVID-19, Mekkawy and colleagues described peripheral nervous manifestations in 29.04% of patients early from the 1st day to 7 days of infection and late from the 8th to the 15th day of affection. Muscle injury was present in 1.37% of patients, and symmetrical lower limb distal sensory neuropathy in 1.55% of the patients [22].

Bagnato and colleagues evaluated 21 patients after COVID-19 infection; neuromuscular affection was demonstrated in 17 patients. Affection varied between critical illness myopathy (CIM), critical illness polyneuropathy (CIP), Guillain-Barré syndrome, and peroneal nerve injury [23].

## Conclusion

Neurological affection including peripheral neuropathy associated with SARS-COV-2 should not be overlooked. Affected personnel may be limited in number but during a massive pandemic, this can have a tremendous effect. Careful short-term neurological assessment is needed for symptomatic patients and can result in early detection and appropriate management.

### Limitations

Long-term follow-up can be done in the future to identify the long-term neurological effects of SARS-COV-2.

## Data Availability

The datasets used and/or analyzed during the current study are available from the corresponding author upon reasonable request.

## Abbreviations

ACE-2: Angiotensin-converting enzyme 2
COVID-19: Coronavirus disease 2019
CRP: C-reactive protein
CMAP: Compound motor action potential
EMG: Electromyography
MOH: Ministry of Health
MNC: Motor nerve conduction
MNCA: Motor nerve conduction amplitude
MNCL: Motor nerve conduction latency
MNCV: Motor nerve conduction velocity
RT-PCR: Reverse transcription-polymerase chain reaction
SARS‐COV‐2: Severe acute respiratory syndrome coronavirus 2
SNC: Sensory nerve conduction
SNCA: Sensory nerve conduction amplitude
SNCL: Sensory nerve conduction latency
SNCV: Sensory nerve conduction velocity
SPO_2_: Saturation of peripheral oxygen
SPSS: Statistical Package for Social Sciences
